# Infrared Nanospectroscopy of Individual Extracellular Microvesicles

**DOI:** 10.3390/molecules26040887

**Published:** 2021-02-08

**Authors:** Raffaella Polito, Mattia Musto, Maria Eleonora Temperini, Laura Ballerini, Michele Ortolani, Leonetta Baldassarre, Loredana Casalis, Valeria Giliberti

**Affiliations:** 1 Department of Physics, Sapienza University of Rome, Piazzale Aldo Moro 2, I-00185 Roma, Italy; raffaella.polito@uniroma1.it (R.P.); maria.eleonora.temperini@roma1.infn.it (M.E.T.); michele.ortolani@uniroma1.it (M.O.); leonetta.baldassarre@uniroma1.it (L.B.); 2 International School for Advanced Studies (SISSA), I-34136 Trieste, Italy; mattia.musto@iit.it (M.M.) laura.ballerini@sissa.it (L.B.); 3 Elettra-Sincrotrone Trieste S.C.p.A., Area Science Park, I-34149 Trieste, Italy; loredana.casalis@elettra.eu; 4 Istituto Italiano di Tecnologia, Center for Life NanoScience, Viale Regina Elena 291, I-00161 Roma, Italy

**Keywords:** extracellular vesicles, infrared nanoscale spectroscopy, atomic force microscopy

## Abstract

Extracellular vesicles are membrane-delimited structures, involved in several inter-cellular communication processes, both physiological and pathological, since they deliver complex biological cargo. Extracellular vesicles have been identified as possible biomarkers of several pathological diseases; thus, their characterization is fundamental in order to gain a deep understanding of their function and of the related processes. Traditional approaches for the characterization of the molecular content of the vesicles require a large quantity of sample, thereby providing an average molecular profile, while their heterogeneity is typically probed by non-optical microscopies that, however, lack the chemical sensitivity to provide information of the molecular cargo. Here, we perform a study of individual microvesicles, a subclass of extracellular vesicles generated by the outward budding of the plasma membrane, released by two cultures of glial cells under different stimuli, by applying a state-of-the-art infrared nanospectroscopy technique based on the coupling of an atomic force microscope and a pulsed laser, which combines the label-free chemical sensitivity of infrared spectroscopy with the nanometric resolution of atomic force microscopy. By correlating topographic, mechanical and spectroscopic information of individual microvesicles, we identified two main populations in both families of vesicles released by the two cell cultures. Subtle differences in terms of nucleic acid content among the two families of vesicles have been found by performing a fitting procedure of the main nucleic acid vibrational peaks in the 1000–1250 cm^−1^ frequency range.

## 1. Introduction

Living cells release in the extracellular environment different types of membrane-enclosed structures generally named extracellular vesicles (EVs), featuring an important role in a variety of inter-cellular communication processes [1,2,3,4,5]. EVs are often classified as apoptotic bodies (ABs) [6], microvesicles (MVs) [7], and exosomes (EXOs) [8], depending both on the way they are originated inside the cells (EXOs) or from their membrane walls (MVs), and on their typical dimensions, ranging from few tens of nanometers for EXOs to several hundreds of nanometers for ABs and MVs [9]. EVs biogenesis pathways indeed may differ according to the producing cell type. On the other side, the biogenesis of both EXOs and MVs share common intracellular mechanisms, that make the possibility of distinguish among different EVs subclasses extremely challenging [2]. Both MVs and EXOs act as vehicles of messenger RNA, proteins and bioactive lipids, constituting an important way of transferring information from one cell to another. As such, they represent a major frontier in pharmacology and in early medical diagnosis studies [10]. Several approaches have been proposed for the characterization of EVs produced by living cell cultures in order to understand their specific functions: their size distribution is measured by means of nanoparticle-tracking analysis and dynamic light scattering [11,12,13]; their average molecular profile is determined through either infrared (IR) or mass spectroscopy approaches, while the RNA content is characterized and quantified mostly by RT-qPCR [14,15,16]. In this framework, IR vibrational spectroscopy can provide direct access to specific molecular content of the EVs, through the study of their specific spectral signatures. Several papers have debated the potential of IR spectroscopy also for EV-based diagnostic approaches [17,18]. Moreover, it has been shown that by combining IR spectroscopy with Principal Component Analysis, it is possible to separate clearly the spectra from EVs extracted in different conditions [19,20].

Unfortunately, due to the nanoscale dimensions of EVs, the sophisticated procedures required for their isolation from the culture medium do not always provide pure ensembles of EVs of the same kind. Therefore, characterization of individual EVs in the ensemble is recommended in addition to the measurements performed on large ensembles of EVs. Single-EV characterization approaches can give insights about the heterogeneity of the EV ensemble, insofar as validating average values from biochemical assays of the same ensemble. In most EV studies, microscopy images of single EV constitute a necessary benchmark for their classification and for understanding their function [21]. Because of the size of EVs being below the diffraction limit for visible-light microscopy, single-EV images are usually obtained with non-optical microscopy techniques such as, e.g., scanning electron microscopy (SEM) [22] and atomic force microscopy (AFM) [23,24,25] that, however, do not have the capability to retrieve information on the molecular composition of the single EVs [26].

Along the line of reasoning of combining vibrational spectroscopy with single EV characterization, AFM-based IR nano-spectroscopy techniques represent a very promising approach, as also demonstrated by the recent results of Kim et al. [27]. IR nano-spectroscopy techniques have, indeed, recently allowed the identification of the chemical composition of nanostructures through the measurement of vibrational spectra of their constituent molecules [28,29]. The added feature of these approaches is that it becomes possible to correlate the mechanical and morphological properties to the molecular content via the IR spectra [30]. This could help to bridge the gap between the spectroscopic study of ensembles of EVs and experimental approaches providing information on individual EVs.

In this work, we concentrated on MVs, which are known for mediating glial cells-neuron communication and for maintaining Central nervous system (CNS) functions [31]. We present an AFM-based IR nano-spectroscopy study of MVs released by two cultures of glial cells in which different types of stimuli were provided to the cells: (i) a conventional MV-release method based on the addition to the cell culture medium of benzoyl-adenosine triphosphate (bzATP); (ii) the exposure of the same cell cultures to graphene oxide (GO) nano-flakes, an innovative non-pharmacological but rather physical method to trigger the release of MVs [32,33]. The study of graphene-based nanomaterials as method to release MVs is motivated by the potential use of either MVs and graphene in the field of drug and gene delivery [34]. In perspective, the advantage of such approach would be to regulate the release of MVs featuring the same biological and mechanical properties as those released physiologically by cells, without affecting recipient cells vitality. Such released MVs might eventually carry GO nano-flakes, previously functionalized to deliver drugs or genes of interest, and then acting both as trigger and a cargo. We use the AFM-IR technique that provides IR vibrational absorption spectrum by monitoring the absorption-induced local thermal expansion of the sample, which is not limited by diffraction [35,36]. Such technique can also provide information on the stiffness of the sample that is measured as a function of the modification of the resonance frequency of the cantilever in contact with the sample. We report that, in the two MV populations, it is possible to identify both MVs that are homogeneous from the morphological and mechanical point of view, and other MVs that show the presence of softer areas, visible also in the topography. These sub-structures display intense protein absorption bands, that are instead negligible in the other MVs. By selectively choosing MVs that are homogeneous within the two populations, we analyze the spectroscopic response of the two sets of MVs in the 1000–1300 cm^−1^ frequency range (wavelengths from 8 to 11 microns), where the main IR vibrational absorption peaks of nucleic acids are found [37,38]. We show that it is possible to distinguish the two populations of MVs in terms of their nucleic acid content.

## 2. Results and Discussion

### 2.1. Nanoimaging and Nanospectroscopy of Microvesicles

**Figure 1:**
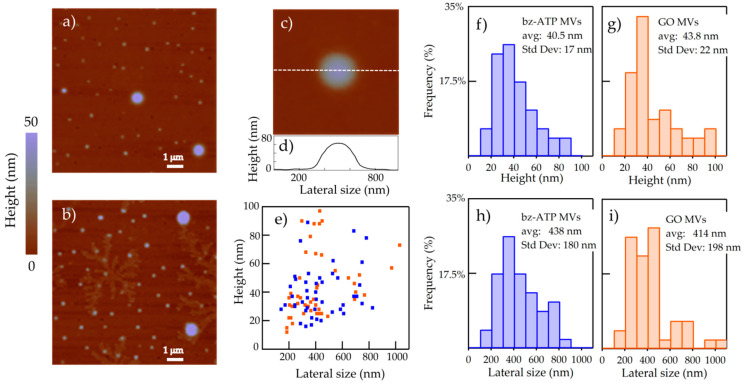
(**a**) Topography map of MVs deposited on ultraflat gold substrate measured by AFM. The image is representative of sample areas where MVs are not contaminated by salt crystals and other residues of the drop-casting process. (**b**) Topography map of MVs deposited on ultraflat gold substrate contaminated by salt crystals. (**c**) Topography map of a single MV deposited on ultraflat gold substrate. (**d**) Line-scan profile taken along the MV from which the height and the lateral size are determined. (**e**) The height vs. lateral size plot for all MVs measured by AFM. (**f**–**i**) Histograms of all measured topographic values, with average and standard deviation indicated in the boxes.

The samples investigated by AFM-IR consist of MVs released by glial cells under two different stressors: (i) benzoyl-adenosine triphosphate (bzATP) and (ii) graphene oxide (GO) nano-flakes- drop-casted and dried on ultraflat gold surfaces. A good adhesion of MVs to the gold substrate is necessary for a proper contact-mode operation mode of the AFM-IR technique, and the deposition of a large amount of individual MVs on the gold substrate increases the statistical significance of the study (see Figure 1a for a topography map of a representative 10 × 10 μm area of one of the chips where several individual MVs of different size are present). We first performed extensive AFM topography of the chips that, besides providing an insight on the homogeneity of the drop-casted samples, also allows us to identify those areas where the MVs are isolated from large salt crystals, such as those reported in Figure 1b, which originate from water evaporation of the suspension containing the MVs and of the buffer solution used to rinse the samples. This is a crucial advantage provided by AFM-IR compared to far-field IR spectroscopies. Indeed, studies of standard Fourier-transform IR spectroscopy (FTIR) and attenuated total reflection FTIR spectroscopy (ATR-FTIR) performed on dry EVs deposited on solid substrates limit the spectroscopic analysis to the protein and lipid absorption peaks (1400–1800 cm^−1^ range), because the unavoidable IR absorption signal in the 1000–1250 cm^−1^ frequency range of phosphate salt crystals could mask the vibrational peaks of nucleic acids [14,17]. Even if the formation of phosphate crystal salts could be circumvented, or their effect could be subtracted from the IR absorption spectra, still the isolation of EVs from other non-EV carriers, e.g., cell membrane flakes containing proteins, lipids and nucleic acids, cannot be achieved in far-field IR spectroscopy, therefore the contribution of EVs may still be masked in the spatially-averaged IR spectra acquired on large sample areas. In this framework, the AFM-based platform could make the IR spectroscopy a method of choice to also determinate the nucleic acids content of MVs due to its capability to measure individual and isolated MVs far from phosphate salt crystals or other non-EV carriers with nanometric resolution. The identification of MVs by AFM can be done from topography data (circular symmetry, diameter and height within specified range) and nano-mechanical data (stiffness comparable to that of a cell membrane) [23,25]. In this respect, the AFM-based IR spectroscopy platform guarantees a very high degree of sample purity for IR spectroscopy, because ideally the spectrum of a single MV with no contributions from surrounding salts and deposition residues can be detected.

In Figure 1c,d, we report the AFM topography map of an individual MV and the corresponding line-profile taken along the center of the MV as example of the typical shape of MVs in our samples. We did not observe significant topography differences between the bzATP-MVs and those released by cultures treated with GO. In both populations, MVs display an almost circular lens-shape: the transformation from a spherical shape of the MVs in solution to a lens-shape when the MVs are dried on substrate chips is probably due to the preferential adhesion of the phospholipidic membrane of the MVs to the ultraflat clean gold surface with hydrophilic behavior and to the minimization of the MV volume in a dry environment.

The AFM topography data were measured on approximately 100 MVs per each group. The data were statistically analyzed and the results are reported in Figure 1e–i. The observed height ranges between 10 and 100 nm, and the lateral size between 100 and 1100 nm. There is weak correlation between height and lateral size pointing to different drying mechanisms for each individual MV (see Figure 1e). The two families are not significantly different as far as the topography parameters are considered; however, the GO-MVs are slightly higher and have smaller lateral size than the bz-ATP ones. The curvature radius of the initial spherical shape of the MVs in solution may be roughly estimated from the lateral size and it falls in the 100 to 1000 nm range; therefore, we can classify these bodies as predominantly MVs [4].

Beside the morphological analysis, the AFM-IR platform exploiting the photo-thermal expansion of samples resulting by IR radiation absorption can provide simultaneously label-free information on the single-MV molecular content. The AFM-IR nanospectroscopy technique provides the IR absorption spectra of the MVs upon monitoring the cantilever oscillations induced by the sample photo-expansion. The cantilever oscillation amplitude Δ*z* can be modeled as:

(1) $$∆z\left(\omega \right)\propto \alpha_{sample}\left(\omega \right)f_{e.m.}I_{laser}Q$$

where *α_sample_* is the sample absorption coefficient, *f_e.m._* is the field enhancement below the AFM tip, *I_laser_* is the IR light intensity and *Q* is the quality factor of the mechanical resonance of the cantilever. The presence of a gold substrate, together with a gold-coated AFM tip, provides a strong enhancement of the optical intensity in the tip-substrate nanogap, enabling to measure the IR-induced thermal expansion of very thin samples, down to molecular monolayers as it has been recently demonstrated [39,40,41]. Under the assumption that *f_e.m._* is constant, it follows that Δ*z* normalized by the IR light intensity is proportional to the sample absorption coefficient *α_sample_*. The AFM-IR setup allows for the acquisition of both local absorption spectra from the sample portion under the tip [35] and maps at fixed IR frequencies [36]. By maintaining the AFM tip fixed at a sample point and sweeping the QCL frequency *ω* one can indeed obtain the local photoexpansion spectrum that, according to Equation (1), ultimately depends only on *α_sample_*(*ω*). Alternatively, one can select an IR frequency emitted by the QCL, which corresponds to a specific vibrational absorption peak of the sample, and making the AFM tip scanning on the sample surface so as to acquire the photothermal expansion pixel by pixel.

**Figure 2:**
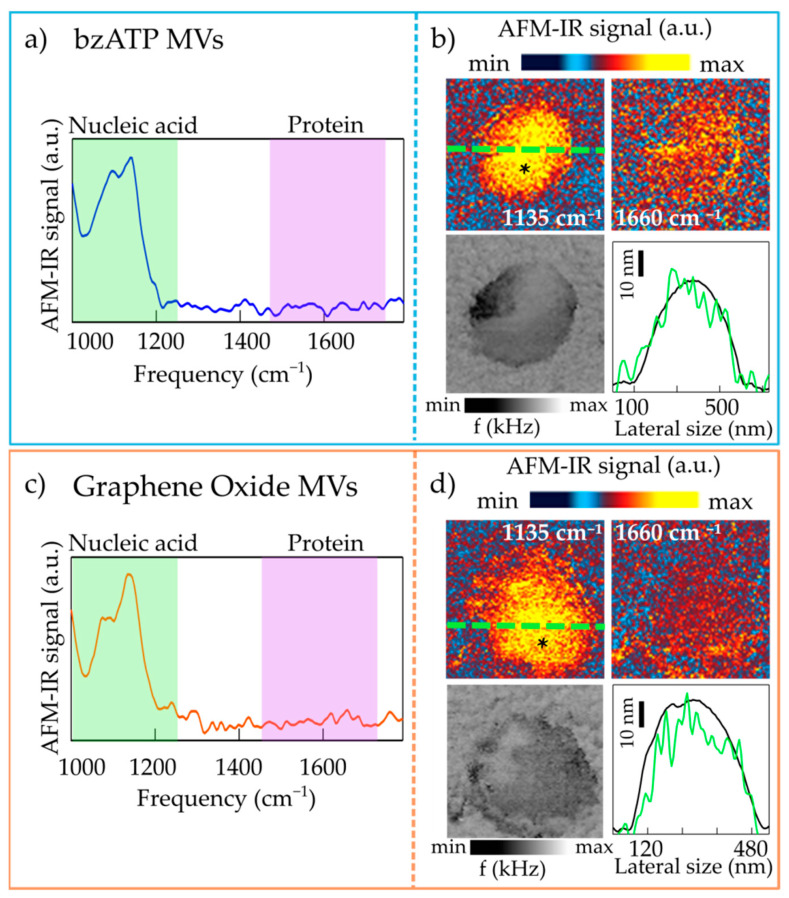
(**a**) AFM-IR spectrum obtained on a representative circular lens-shaped bzATP-MV; the asterisk in the map of panel (**b**) marks the point where the spectrum has been acquired. (**b**) Top: AFM-IR maps acquired at 1135 cm^−1^ (left) and at 1660 cm^−1^ (right) on the bzATP-MV. Bottom left: map of the mechanical resonance frequency *f*. Bottom right: topography line-scan profile (black curve) and AFM-IR signal line-scan acquired at 1135 cm^−1^ (green curve), taken along the green dashed line in the upper-left sub-panel. (**c**,**d**) Same as (**a**,**b**) for a representative circular lens-shaped GO-MV.

In Figure 2, we report both the IR maps and the spectra acquired on representative bzATP-derived MVs and GO-derived ones deposited on ultraflat gold substrate. Both curves of Figure 2a,c display a structured absorption band at about 1100 cm^−1^, mainly composed of two peaks: (i) one centered at 1080 cm^−1^ and a (ii) second one around 1135 cm^−1^.

Absorption peaks originating from the vibrations of PO2 bonds of the nucleic acid backbone are expected to fall in the 900–1300 cm^−1^ spectral range, with the symmetric stretching mode expected around 1080 cm^−1^ and the antisymmetric mode centered between 1215 and 1245 cm^−1^ [37,38]. In the case of RNA only, the presence of the hydroxyl group attached to the furanose ring in the 2’ position leads to additional peaks arising from the stretching of the C1’C2’OC3’ structure [37,38] and expected to fall in between the PO2 symmetric and antisymmetric modes. Peaks related to PO2 bonds and CO bonds present in the phospholipid heads should also be present in the spectra in this spectral region. However, in the single-MV AFM-IR spectra in Figure 2a,c, there are no absorption peaks corresponding to those attributed to lipids molecules and found in the ATR-FTIR spectra [14] at 1390, 1450 cm^−1^ (CH bending mode) and 1740 cm^−1^ (C=O stretching of the lipid ester bonds) [42]. Therefore, we can conclude that, since we do not observe a photo-expansion signal from lipids probably because of thermo-mechanical reasons, the IR absorption peaks in the 1000–1300 cm^−1^ range can be almost entirely related to nucleic acids. In particular, the peak centered around 1080 cm^−1^ can be safely assigned to the strong symmetric PO2 stretching vibration, while the peak centered around 1135 cm^−1^ is related to the absorption vibrations arising from the stretching of the C1’C2’OC3’ structure in RNA. The PO2 antisymmetric mode is not visible as a distinct peak in the AFM-IR spectra of the MVs; however, one can speculate that it contributes to the high-frequency tail of the peak at 1135 cm^−1^ [37,38]. The small dipole moment of this vibrational peak could prevent the identification of such mode, as verified also in AFM-IR spectra of RNA control samples, studied in ref. [43] where there is no ambiguity on chemical content of samples. In the region between 1400 and 1700 cm^−1^ no well-defined peaks are seen. Usually in this region one expects the main absorption peaks related to C=O stretching (amide-I band) and N-H bending (amide-II band) of the protein backbone [44]. We note that this does not necessarily imply the absence of proteins in those MVs, but rather a protein concentration below the detection threshold of our AFM-IR experiment, which leads to unobservable photo-thermal expansion. This is confirmed also by the nano-imaging experiments performed at 1135 and 1660 cm^−1^, assumed as the center frequencies of the absorption peaks of RNA and of amide-I of proteins, respectively. In Figure 2c,d, one can indeed notice an homogeneous strong AFM-IR signal at 1135 cm^−1^, that is instead almost negligible at 1660 cm^−1^. Together with the AFM-IR absorption maps we report also the map of the frequency of the mechanical resonance of the AFM cantilever in contact with the sample (*f*). The quantity *f* is an indirect probe of sample mechanical properties since the higher the *f*, the higher the sample stiffness [36]. The homogeneity in the composition of the two homogeneous MVs reported in Figure 2 also appears in the *f* maps that do not show softer or harder areas within the sample. The quantity *f* can be used to evaluate the photothermal expansion force *F*_abs_ from the AFM-IR signal amplitude, using the formulas reported in ref. [39], and then the Young’s modulus E* of the MVs in Figure 2. We obtain E* in the range 20 to 200 MPa by using the measured *F*_abs_ values in the range 0.05 to 0.5 nN. We notice that the large uncertainty of this calculation depends on the uncertainty on the power density of the IR laser impinging on the MV, and on the unknown details of the thermal conduction between the AFM tip and the MV, and between the MV and the substrate.

**Figure 3:**
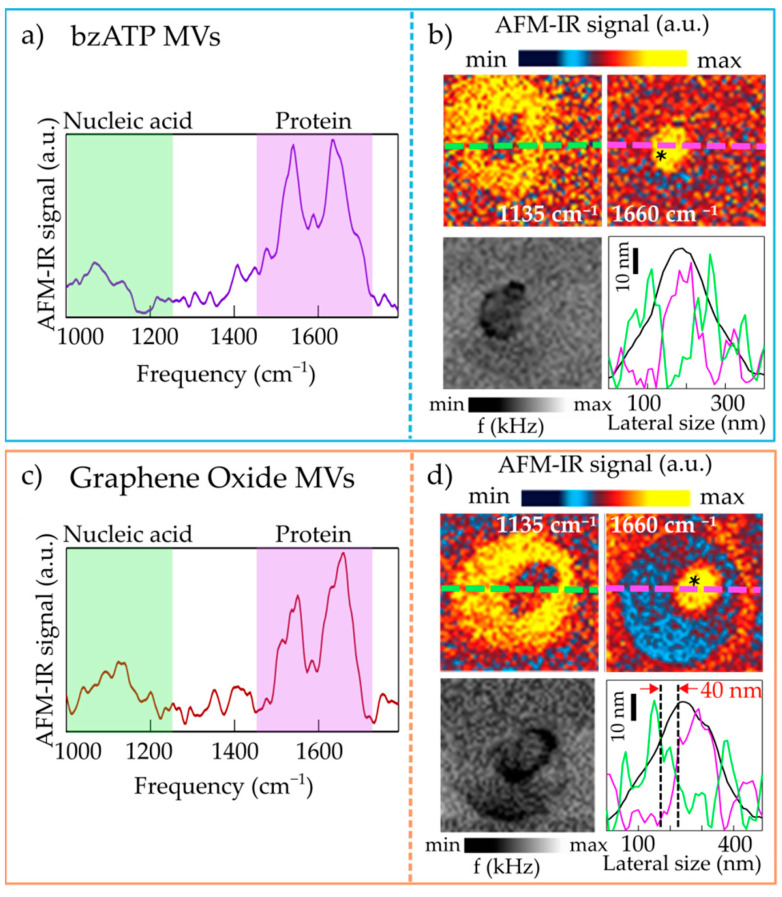
(**a**) AFM-IR spectrum obtained on a representative bzATP-MV featuring heterogeneous mechanical, morphological and spectroscopic properties; the asterisk in the map of panel (**b**) marks the point where the spectrum has been acquired. (**b**) Top: AFM-IR maps acquired at 1135 cm^−1^ (left) and at 1660 cm^−1^ (right) on the bzATP-MV. Bottom left: map of the mechanical resonance frequency *f*. Bottom right: line-scan profiles taken along the MV in the topography map (black curve) and along the AFM-IR map acquired at 1135 cm^−1^ (green curve) and at 1660 cm^−1^ (violet curve). (**c**,**d**) Same as (**a**,**b**) for a representative GO-MV featuring heterogeneous mechanical, morphological and spectroscopic properties. The vertical dotted lines in panel (**d**) (bottom right) indicate a lateral resolution of around 40 nm.

We have occasionally found MVs that display an irregular profile from the morphological point of view, as shown in Figure 3, for both bzATP-MVs and GO-MVs. The irregular line profile originates from the presence of a softer region within the MV. Indeed, the resonance frequency (*f*) maps in Figure 3b,d show a clear decrease in the resonance frequency of the cantilever bending mode. Qualitatively, *F*_abs_ is inversely proportional to *f* [39], so the small decrease in *f* across the bottom-left maps of Figure 3b,d corresponds to a small increase in *F*_abs_, and therefore to a softer material with slightly lower E*. Thanks to the high lateral resolution of AFM-IR it is possible to identify the spectra of these softer regions alone (Figure 3a,c), that display strong amide-I and amide-II protein bands, at odd with the spectra measured on the rest of the MVs (not shown). The AFM-IR maps (Figure 3b,d) show a clear contrast between the softer area that displays strong absorption at 1660 cm^−1^, and the rest of the MV (outer ring) that instead is dominated by the signal at 1135 cm^−1^. We have also measured the entire spectrum of the outer ring (not shown) that is quite compatible with the spectrum of the MV in Figure 2. In the line-scan profiles taken along the maps and reported in Figure 3b,d (bottom panels, green curves for the maps at 1135 cm^−1^ and violet curves for those at 1660 cm^−1^) one sees that a lateral resolution of 40 nm for IR photoexpansion spectroscopy is achieved in our experiment (as indicated by the dotted vertical lines and red arrows in Figure 3d). The high lateral resolution is provided by the strong radiation field enhancement that takes place at the nanogap between the gold-coated AFM tip and the ultraflat gold substrate in the case of very thin samples. Under these conditions, the thermal diffusion length of the sample is negligible due to the strong gradient of optical field intensity between the region just below the AFM tip and the rest of the sample [39,40,41].

Both sets of MVs, therefore, consist mainly of homogenous vesicles that absorb at the frequency of the RNA mode in their entire volume. Some heterogeneous MVs, which also have protein aggregates in their cargo, can be found for MVs obtained with both bzATP and GO stressors.

### 2.2. Nanospectroscopy of RNA in Microvesicles

We performed a spectroscopic characterization, focusing on the low-frequency range, of mechanically homogenous MVs from the two populations, with the aim of addressing any difference in their respective nucleic acids content. We decided to exclude from our analysis those MVs that feature strong protein absorption so as to be sure that the photothermal expansion originates from the absorption of nucleic acids only. Weak protein absorption bands in the low-frequency range may indeed be present in the case of high protein absorption signal [44]. Moreover, one of the greatest advantages of using a nano-spectroscopy approach is that one can select subgroups of the MVs that, featuring similar mechanical and morphological properties, could have in principle similar functional behavior and compare them more clearly.

**Figure 4:**
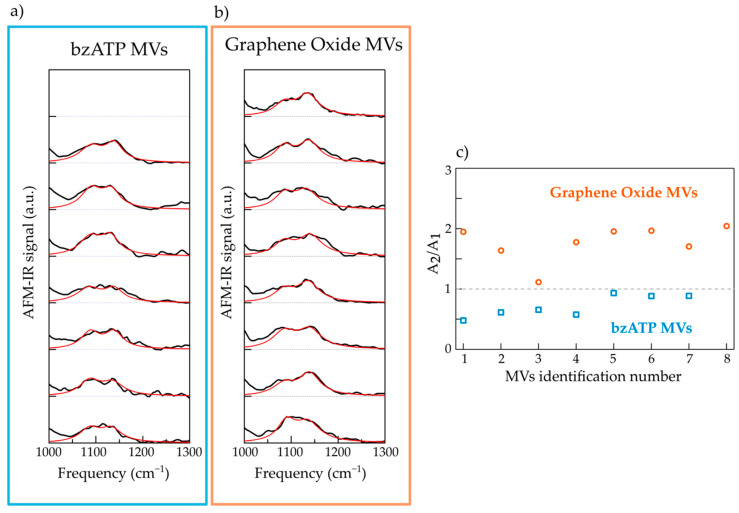
(**a**) AFM-IR spectra (black curves) of 7 individual bzATP-MVs deposited on ultraflat gold substrate with the relative fitting curves (red lines). (**b**) Same as (**a**) for 8 individual GO-MVs. (**c**) Graph of the ratio between the spectral weights of component centered at 1135 cm^−1^ (A2) and that centered at 1085 cm^−1^ (A1).

In Figure 4, we plot in the 1000–1300 cm^−1^ range the AFM-IR spectrum (black curves) of 7 selected MVs of the bzATP type (panel a) and of 8 selected MVs of the GO type (panel b). All the spectra reveal two main strong bands, one centered around 1135 cm^−1^ and another one at lower frequency, which, as discussed above, can be mainly attributed to a stretching mode of RNA and to the PO2 symmetric mode of nuclei acids, respectively. The presence of these two peaks in all the spectra confirms a RNA cargo in all the analyzed MVs, regardless of the specific cell culture.

In order to investigate possible differences in terms of the chemical content of the cargo of the two populations of MVs, we fitted the two absorption bands, i.e., in the 1030–1200 cm^−1^ range, with the sum of two lorentzian contributions. The spectra were normalized to the maximum of the low-frequency band so as to compare the relative intensity of the two contributions regardless the enhancement of the signal at the tip-substrate nanogap, which strongly depends on the MV thickness. The frequency of the two lorentzians are peaked at *v*_1_ = 1085 cm^−1^ and *v*_2_ = 1135 cm^−1^, while the width and the intensity are left as free-fitting parameters. The value of the width of the two contributions varies between 45 and 60 cm^−1^.

The best fit to the sum of the two lorentzian components are reported in Figure 4a,b as red curves. In some cases, the best fit curves do not perfectly reproduce the experimental data and this can be assigned to the presence of other weak absorption peaks that result in shoulders of the main bands and/or broadening of them. However, even a simple fitting model based on two contributions only allows to distinguish the two populations of MVs. This is clear in Figure 4c where we plot, for the two sets of spectra, the distribution of the ratio between the spectral weight of the component centered at 1135 cm^−1^ (A_2_) and that centered at 1085 cm^−1^ (A_1_). One can observe that, for all selected MVs of the bz-ATP type the ratio value A_2_/A_1_ is less than 1, while for all selected MVs of the GO type A_2_/A_1_, it is greater than 1. These results point to a difference among the two sets in terms of nucleic acid content, probably suggesting the coexistence of RNA and other absorbent chemical species in the MVs of the bz-ATP type. One possibility should be the presence of a small amount of DNA, which indeed would justify the decrease in the relative weight of the peak uniquely attributed to RNA (peak centered at 1135 cm^−1^) compared to the one of PO2 vibrational mode (peak at 1085 cm^−1^), that is present in all nucleic acids. However, in order to validate this hypothesis it is necessary to investigate, with further intensive studies, the spectral region at very low-frequencies (less than 1000 cm^−1^) where DNA spectral markers are present, that suffers from low signal to noise ratio, due to a low peak power emission from our IR laser.

## 3. Materials and Methods

### 3.1. Sample Preparation: Cell Culture and MVs Isolation

MVs are EVs generated by the outward budding and fission of the plasma membrane, and they feature typical dimensions between 100 and 1000 nm.

#### 3.1.1. Glial Cultures

Primary glial cultures were prepared from neonatal rat (Wistar) cortices at postnatal day 2–3 as previously described [32,45]. Briefly, dissociated cells were plated and cultured for 3 weeks in 150 cm^2^ flasks, at 37 °C; 5% CO_2_ in culture medium composed of DMEM (Invitrogen) and supplemented with 10% fetal bovine serum (FBS; Thermo Fisher), 100 IU/mL penicillin, and 10 mg/mL streptomycin.

#### 3.1.2. MVs Isolation

MVs were harvested from glial cultures medium and isolated by differential centrifugations, as previously described in Rauti et al. [32]. MVs of GO experimental group were obtained from glial cultures previously exposed to graphene oxide nanoflakes, added once to culture medium and left for 6 days. At the end of the 6-day exposure, culturing medium was removed and standard extracellular solution was added for 1 h, in order to collect the released MVs. The other pool of MVs was instead obtained from glial cultures treated with bzATP, added to standard extracellular solution replacing the culturing medium, for 30 min.

### 3.2. Sample Preparation for AFM-IR Analysis

Droplets of less than 2 μL of the two suspensions containing respectively bzATP-MVs and GO-MVs were deposited on ultraflat template-stripped gold substrate chips (by Platypus Technologies, 0.3 nm rms roughness) and let dry in air. After 10 min, the chips were rinsed with a buffer solution (phosphate buffered saline at pH = 7.8) and subsequently dried for 1 h in an atmosphere with humidity below 20%.

### 3.3. AFM Topography

Topographic height images were recorded at 200 × 200 pixels at a scan rate of 0.5 Hz and resolution of 5 nm (image size 1 *×* 1 microns). We used gold-coated silicon cantilevers (provided by Anasys Instruments, model “PR-EX-NIR2-10 for NanoIR2”) with free resonant frequency of 13 kHz and spring constant between 0.07 and 0.4 N/m. The curvature radius of AFM tips apex is around 25 nm as confirmed by electron microscopy imaging. Image processing was performed using Analysis Studio analysis software (Anasys Corp., Santa Barbara, CA, USA). The dimensions of each vesicle were evaluated from cross-line profiles (height as the max value, lateral size as twice the full-width at half maximum of the bell-shaped profiles), and the results were statistically analyzed using Igor Pro, version 6.0.

### 3.4. AFM-IR Nanospectroscopy

The AFM-IR platform (NanoIR2 by Anasys Instruments) is based on the coupling of the AFM system with a TM-polarized external-cavity mid-IR quantum cascade laser (QCL-MIRcat-PX-B by Daylight Solutions) focused on the tip-sample system with an incident angle of 70 degrees. The mid-IR QCL parameters were set at 160 ns for the pulses width and around 200 kHz for the repetition rate such as to be in resonance with the second bending mode of the cantilever. Metal-mesh filters in front of QCL output ensured that laser power on the sample does not exceed 10% of the maximal one (~500 mW) in order to avoid signal saturation and/or damage of sample.

The AFM-IR spectra were collected with a spectral resolution of 4 cm^−1^ within the range 1000–1800 cm^−1^. The curves reported in Figure 2, Figure 3 and Figure 4 have been obtained by normalizing the AFM-IR spectrum acquired with the AFM tip located on top of the MV by the AFM-IR spectrum acquired, under the same experimental conditions, on an area of bare and clean ultraflat gold surface so as to remove the photo-expansion of the gold support and of the gold-coated AFM tip. Successively, they were smoothed with a spline algorithm. Finally, all spectra in Figure 4 were normalized to the PO_2_ symmetric mode absorption at 1085 cm^−1^.

## 4. Conclusions

In conclusion, we have demonstrated that AFM-IR represents a non-destructive single-MV approach that enables to combine biochemical, structural and mechanical information on individual MVs. In particular, we conducted experiments on MVs released by two cultures of glial cells, a first one where a conventional method based on the addition of benzoyl-adenosine triphosphate (bzATP) was adopted, and a second one where, instead, the same cell culture was exposed to graphene oxide (GO) nano-flakes. By combining topographic, mechanical and spectroscopic information of individual MVs we found that both populations of MVs (bzATP and GO) consist mainly of homogeneous vesicles that absorb at the stretching mode of RNA at 1135 cm^−1^ in the entire volume, while few heterogeneous MVs display a protein aggregate in their cargo. The AFM-IR spectroscopic analysis of the homogeneous MVs points to possible subtle differences between the two populations in terms of nucleic acid content. Given the potential use of MVs in drug delivery applications, it would be advantageous to regulate MV release through cell exposure to different types of graphene-based nanomaterials, insofar as generating vesicles, with well-defined cargo, that have the same biological and mechanical properties as those released physiologically by cells. In perspective, AFM-IR could be applied to many kinds of extracellular vesicles including EXOs and ABs.

## Data Availability

Data is contained within the article.

## Sample Availability

Samples not available.
